# Radiosensitization in Pediatric High-Grade Glioma: Targets, Resistance and Developments

**DOI:** 10.3389/fonc.2021.662209

**Published:** 2021-04-01

**Authors:** Dennis S. Metselaar, Aimée du Chatinier, Iris Stuiver, Gertjan J. L. Kaspers, Esther Hulleman

**Affiliations:** ^1^ Department of Neuro-oncology, Princess Máxima Center for Pediatric Oncology, Utrecht, Netherlands; ^2^ Emma Children’s Hospital, Amsterdam UMC, Vrije Universiteit Amsterdam, Pediatric Oncology, Cancer Center Amsterdam, Amsterdam, Netherlands

**Keywords:** pediatric high-grade glioma (pHGG), radiotherapy, glioma, radio-enhancement, radiosensitizer, radioresistance

## Abstract

Pediatric high-grade gliomas (pHGG) are the leading cause of cancer-related death in children. These epigenetically dysregulated tumors often harbor mutations in genes encoding *histone 3*, which contributes to a stem cell-like, therapy-resistant phenotype. Furthermore, pHGG are characterized by a diffuse growth pattern, which, together with their delicate location, makes complete surgical resection often impossible. Radiation therapy (RT) is part of the standard therapy against pHGG and generally the only modality, apart from surgery, to provide symptom relief and a delay in tumor progression. However, as a single treatment modality, RT still offers no chance for a cure. As with most therapeutic approaches, irradiated cancer cells often acquire resistance mechanisms that permit survival or stimulate regrowth after treatment, thereby limiting the efficacy of RT. Various preclinical studies have investigated radiosensitizers in pHGG models, without leading to an improved clinical outcome for these patients. However, our recently improved molecular understanding of pHGG generates new opportunities to (re-)evaluate radiosensitizers in these malignancies. Furthermore, the use of radio-enhancing agents has several benefits in pHGG compared to other cancers, which will be discussed here. This review provides an overview and a critical evaluation of the radiosensitization strategies that have been studied to date in pHGG, thereby providing a framework for improving radiosensitivity of these rapidly fatal brain tumors.

## Introduction

Cancer is one of the leading causes of death among children in developed countries. Among pediatric cancers, central nervous system (CNS) tumors represent the second-most common and the most lethal group, accounting for around 40 percent of cancer-related deaths (1). While the prognosis of children with almost all types of cancer has improved over the past decades, this improvement is minimal in children with CNS tumors (2). This dismal prognosis is mainly caused by pediatric high-grade gliomas (pHGG); aggressive tumors that often originate from glial progenitor cells in the CNS (3–5). pHGG comprise all pediatric glioma lesions that are classified as ‘grade III’ or ‘grade IV’ by the World Health Organization (WHO) (6). A subset of pHGG, referred to as diffuse midline glioma (DMG) (formerly known as diffuse intrinsic pontine glioma or DIPG), arise in the midline of the brain and carry a particularly grim prognosis (5, 7). Children with DMG have a median survival of 11 months, with less than 1 percent surviving past 5 years after diagnosis (8, 9). Glioblastoma (formerly known as glioblastoma multiforme) are the most common subset of pHGG and have a reported 5-year survival rate of less than 20 percent (10).

In recent years, distinct pHGG entities have been identified based on recurrent mutations affecting the epigenome. One entity is characterized by a missense lysine-to-methionine substitution at amino acid 27 of the tail of histone H3.1 or H3.3 (H3-K27M) (11). Another pHGG subgroup is characterized by glycine-to-arginine/valine substitutions at amino acid 34 in histone H3.3 (H3-G34R/V) and has recently been described as the first identified pHGG with a neuronal rather than glial precursor cell of origin (11–14). These epigenetically mutated entities have a distinct neuroanatomical predilection. K27M mutations occur exclusively in the midline of the brain, while G34R/V mutations occur exclusively in the cerebral cortex (11). Notably, these mutations represent a hallmark characteristic for pediatric versus adult HGG (aHGG), defining ~50 percent of pediatric cases compared to less than 1 percent of adults, emphasizing the necessity to research them independently (15).

pHGG are characterized by a diffuse and infiltrative growth pattern, often in delicate and difficult to reach parts of the brain, which makes complete surgical removal often not an option (3, 16). Gross total resection of diffuse tumors in the midline of the brain is particularly not possible as these tumors are intricately woven into areas of normal neural tissue that control vital functions, such as heart rate and breathing. The standard of care for most midline tumors, except for infants, is fractionated radiation therapy (RT) (7). Although this treatment modality provides temporary symptom relief, a minor delay in tumor progression, and a three-month survival benefit on average, it offers no chance for a cure (3, 16, 17). For diffuse tumors in the cerebral cortex, partial surgical resection is often performed, followed by RT and chemotherapy (4, 16). In addition to the surgical difficulties, pHGG often gain resistance to the applied chemotherapy or the therapy does not reach the tumor at all due to inadequate penetration of the blood-brain barrier (BBB) (18). As a result, pHGG are still among the most lethal tumors in children and improved therapeutic options are desperately needed.

Ionizing radiation essentially impairs tumor growth by evoking DNA damage, either directly or through reactive oxygen species (ROS). In response to DNA damage, cell cycle checkpoint kinases are alerted to initiate DNA damage response (DDR) in which cell cycle progression is halted and the DNA-repair machinery is activated (19). The ability of DDR proteins to sense DNA damage and activate repair pathways play an essential role in regulating radiation sensitivity, because the amount of DNA damage is a critical factor for the therapeutic efficacy of RT (20). As a resistance mechanism, irradiated cancer cells often increase their DNA-repair efficiency by enhancing the expression of DDR components (21). In addition, as with tissue injury at large, RT-induced cytotoxicity typically activates mitogenic signaling pathways, resulting in an enhanced proliferation rate and repopulation of the tumor volume (21). To improve the sensitivity of tumor cells to radiation, various studies have investigated compounds that can counteract these resistance mechanisms or enhance the radiation effect in a different manner. These compounds are referred to as radiosensitizers and are defined as “*compounds that, when combined with radiation, achieve greater tumor inactivation than would have been expected from the additive effect of each modality*” (22).

**Figure 1:**
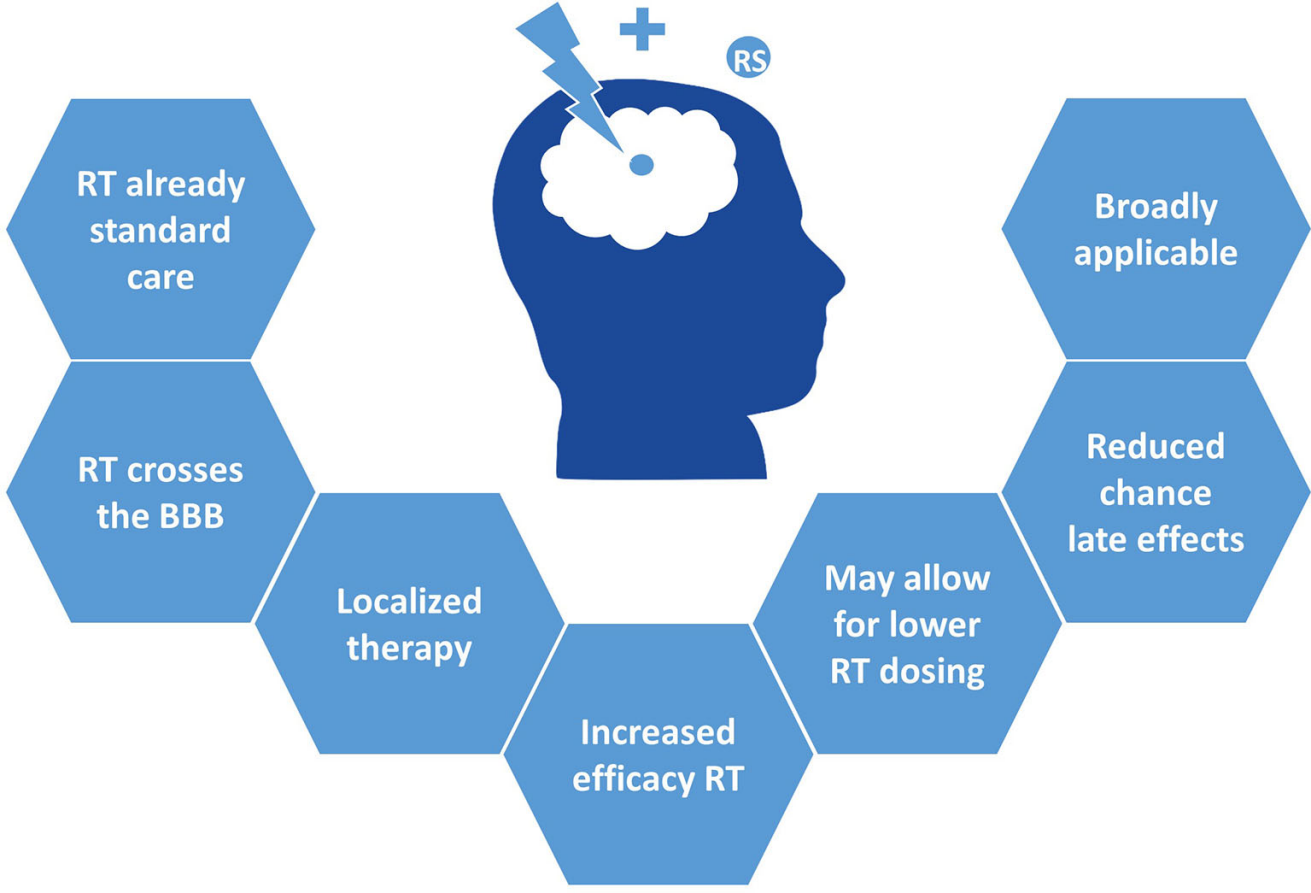
Clinical advantages of radiosensitizers in pHGG.

This concept of radiosensitization is of particular interest in pHGG, where radiosensitizers may increase the efficacy of RT and thereby allow the use of lower radiation doses to achieve a similar anti-tumor effect, while sparing healthy brain tissue. As such, this could reduce the chance of long-term toxicity and late effects such as neurocognitive dysfunction, growth impairment, and secondary malignancies. Moreover, the risk of added toxicity of such combination therapies is lower, given that the cytotoxic effect of a good radiosensitizer is mainly exploited within the irradiated tumor area. Furthermore, the advantage of drug synergism with RT, instead of drug-to-drug- synergism, is that at least half of the combination is not obstructed by the BBB, thus essentially requiring only one drug to pass this barrier. Finally, radiosensitizers can relatively easily be combined with standard clinical care, as it makes use of the already applied RT. Together, this makes for a broadly applicable approach, and exploring its full potential can contribute considerably to the improvement of current therapy (**Figure 1**).

In this review, we summarize the molecular determinants of radiosensitivity identified in pHGG and provide a critical evaluation of the radiosensitization strategies, and their underlying mechanisms, studied to date. These strategies can be divided into targeting TP53 and protein phosphatase 1D (PPM1D), DNA damage repair, ROS, mitogen-activated protein kinase (MAPK) and phosphoinositide 3-kinase (PI3K) signal transduction pathways, the cell cycle, cancer stem cells (CSCs), and the epigenome. We summarize and discuss the current knowledge on radiosensitization in pHGG and aim to provide researchers and clinicians with leads to further develop (pre)clinical therapy for these rapidly fatal brain tumors.

All preclinical and clinical studies that will be discussed in this review are summarized in **Tables 1** and **2** respectively.

**Table 1:** Overview preclinical radiosensitization studies addressed in this review.

Target	*In vitro* efficacy	*In vivo* efficacy	pHGG model	Remarks	References
**PPM1D**	+	+	H3.3-K27M DIPG	PPM1D-mutant cells more sensitive than PPM1D-WT cells	23
	+	n/a	H3.3-K27M DIPG	Synergy with PARP inhibition PPM1D-mutant cells more sensitive than PPM1D-WT cells	24
**CHK1**	+	n/a	H3.1-K27M DIPG H3.3-K27M DIPG	TP53-mutant cells more sensitive than TP53-WT cells	25
**ATM**	+	n/a	H3.3-K27M anaplastic astrocytoma H3-WT GBM		26
	+	+	H3.3-K27M anaplastic astrocytoma		27
	n/a	+	PDGF-B driven TP53-deficient BSG mouse model	TP53-mutant cells more sensitive than TP53-WT cells	28
**WEE1**	+	+	H3.3-K27M DIPG		29
	+	+	H3-WT GBM H3-G34R GBM H3.3-K27M DIPG		30
**PLK1**	+	n/a	H3.1-K27M DIPG H3.3-K27M DIPG		31
**BUB1/BUBR1**	+	n/a	H3-WT GBM		32
**CDK4/6**	n/a	+	PDGF-B driven Ink4a-ARF-deficient BSG mouse model		33
**Notch**	+	n/a	H3.3-K27M DIPG		34
**PARP**	+	n/a	H3-G34R GBM		35
	+	+	H3-WT HGA H3-G34R HGA H3.3-K27M DIPG		36
**MTH1**	+	n/a	H3-WT GBM H3-G34R GBM	Synergy with PARP inhibition	37
**IGF-1R**	+	n/a	H3-WT GBM H3-G34R GBM		38
**mTOR**	+	n/a	H3.3-K27M DIPG		39
	+	n/a	H3.3-K27M DIPG		40
**PI3K/mTOR**	+	n/a	H3-WT GBM		41
**JMJD3**	+	+	H3.3-K27M DIPG	H3-K27M-mutant cells more sensitive than H3-WT cells	42
	+	n/a	H3.3-K27M DIPG	Synergy with mutant-p53 inhibition	43
**HDAC**	+	n/a	H3-WT GBM H3-G34R GBM		44
	+	n/a	H3.3-K27M DIPG	Synergy with AXL inhibition	45
**PI3K/HDAC**	+	+	H3-WT GBM H3-G34R GBM H3.1-K27M DIPG H3.3-K27M DIPG		46
**BRD4**	+	+	H3-K27M DIPG		47

GBM, glioblastoma multiforme; DIPG, diffuse intrinsic pontine glioma; HGA, high-grade astrocytoma; BSG, brainstem glioma; n/a, data not available.

**Table 2:** Overview clinical radiosensitization studies addressed in this review.

Target	Drug(s)	Population	Study	References
**PARP**	Veliparib	Newly diagnosed DIPG	Phase 1/2	48
**Glutathione S-transferase**	Etanidazole	DIPG	Phase 1	49
**Thioredoxin and ribonucleotide reductases**	Motexafin gadolinium	DIPG	Phase 1	50
	Motexafin gadolinium	DIPG	Phase 2	51
**EGFR**	Erlotinib	HGG	Phase 1	52
	Erlotinib	Brainstem glioma	Phase 1	53
	Gefitinib	Newly diagnosed brain stem gliomas or supratentorial malignant gliomas	Phase 1	54
	Gefitinib	Newly diagnosed brainstem gliomas	Phase 2	55
	Cetuximab	Newly diagnosed DIPG and HGA	Phase 2	56
	Nimotuzumab	DIPG	Phase 2	57
	Nimotuzumab	Newly diagnosed DIPG	Phase 3	58
**VEGF**	Bevacizumab	DIPG/HGG	Retrospective analysis	59
	Bevacizumab	Newly diagnosed DIPG/HGG		60
	Bevacizumab	Newly diagnosed HGG	Phase 2	61
**various RTKs**	Vandetanib	DIPG	Phase 1	62
	Vandetanib and Dasatinib	Newly diagnosed DIPG	Phase 1	63
	Imatinib	Newly diagnosed brainstem and recurrent malignant gliomas	Phase 1	64
**HDAC**	Panobinostat	Progressive DIPG	Case study	65
	Valproic acid	HGG	Retrospective analysis	66
	Valproic acid	DIPG	Retrospective analysis	67
	Valproic acid	Newly diagnosed DIPG or HGG	Phase 2	68
	Vorinostat	Newly diagnosed HGG	Phase 2	69

DIPG, diffuse intrinsic pontine glioma; HGA, high-grade astrocytoma; HGG, high-grade glioma; n/a, data not available.

## TP53 and PPM1D

As with most cancers, the response to RT is not uniform among pHGG patients and appears to be associated with the tumor’s mutational status. Initially, response to RT in H3-K27M pHGG correlates with the type of histone H3 mutation, with patients carrying a *H3F3A* (H3.3) mutation having a significantly worse clinical and radiological response and earlier relapse than those with *HIST1H3B* (H3.1) mutations (70, 71). In contrast, Werbrouck et al. demonstrated that radioresistance is not correlated to the type of H3-K27M mutation but rather driven by alterations of the tumor suppressor TP53, which is a critical component of the DDR downstream of checkpoint kinases (25). The discrepancy between these studies likely stems from the confounding factor that most H3.3-K27M tumors are also TP53-mutant, whereas H3.1-K27M tumors rarely are (72). In order to account for this confounding factor, the latter study performed a multivariate analysis adjusted for age at diagnosis, TP53, and histone H3 mutational status and demonstrated that there was no difference in clinical or radiological response to RT when comparing patients according to H3 mutational status. In contrast, patients carrying a TP53 mutation had a significantly worse clinical and radiological response to RT. At the same time, the type of H3-K27M mutation appeared to be a stronger predictor of post-irradiation relapse and overall survival, whereas TP53 alterations were only marginally associated with survival. As such, these studies suggest that short-term response to RT is driven by TP53 mutations, whereas long-term prognosis after RT is mainly determined by the type of H3-K27M mutation. Since Werbrouck et al. analyzed various other determinants of radiosensitivity on a preclinical and clinical level, the study will be discussed on multiple occasions throughout this review in relation to the corresponding topics.

**Figure 2:**
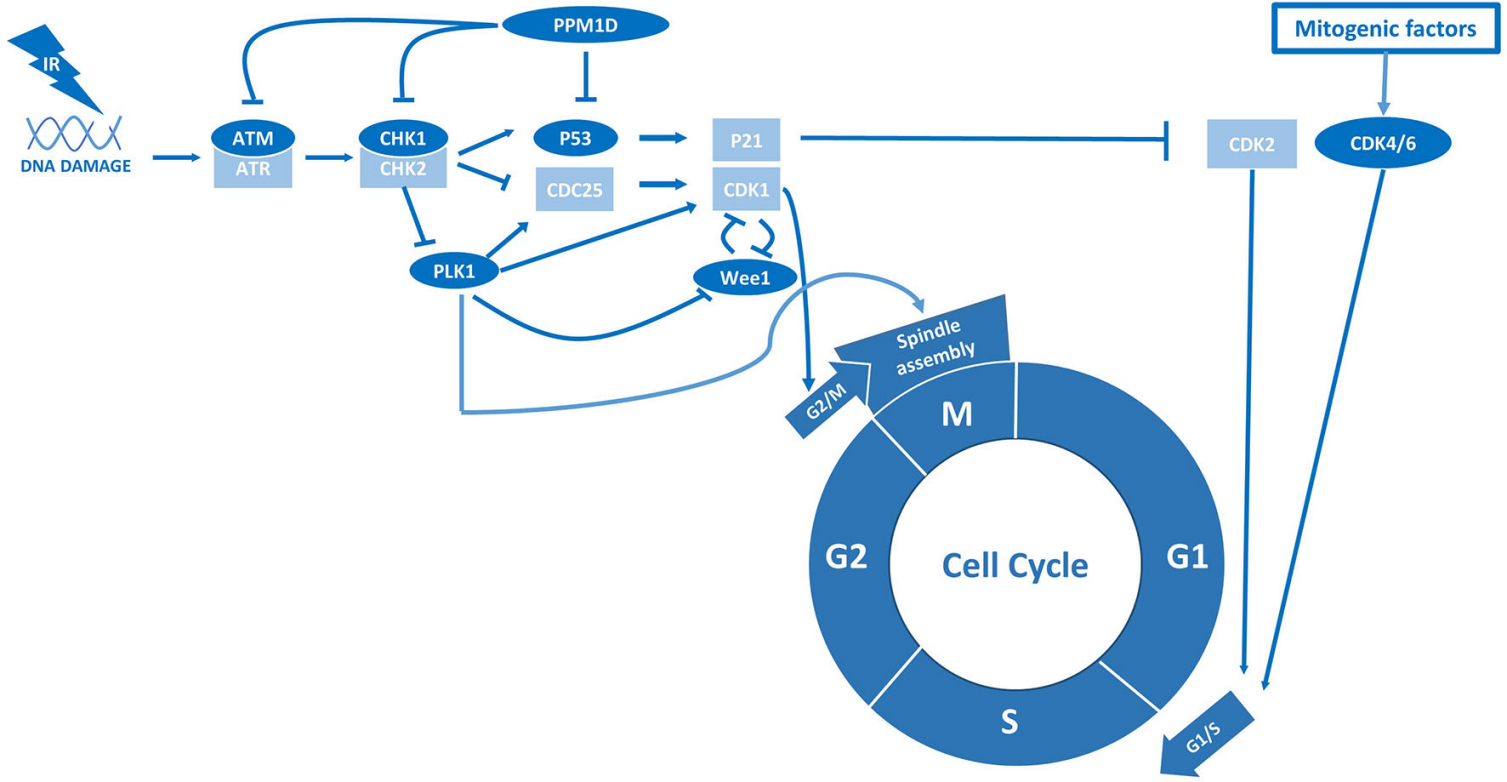
p53 and PPM1D are central regulators of radiation sensitivity in pHGG.

Although the majority of H3.3-K27M tumors harbor a TP53 mutation, a subset of H3.3-K27M, TP53-wildtype tumors contain a gain-of-function mutation in the gene *PPM1D* instead (73). PPM1D encodes the protein wildtype p53-induced phosphatase 1 (WIP1), which dephosphorylates and inactivates p53 (23). Loss-of-function TP53 and gain-of-function PPM1D mutations are mutually exclusive and often considered to be functionally equivalent (73). However, while TP53 alterations are associated with overt radioresistance, PPM1D-mutant tumors appear to have an intermediate radiosensitive phenotype compared to TP53-mutant and -wildtype tumors (23, 25). As a potential explanation for this intermediate phenotype, PPM1D has been shown to affect the DDR independent of its effect on p53 (24). For example, PPM1D inactivates the checkpoint kinases ATM, ATR, and CHK1/2 and consequently impairs the initiation of the DDR after RT (**Figure 2**) (24). Moreover, PPM1D dephosphorylates the protein H2AX and therewith prevents the repair of damaged DNA directly (24). Thus, the enhanced activity of PPM1D that is associated with gain-of-function PPM1D mutations may both reduce radiosensitivity by inhibiting p53 and increase radiosensitivity by reducing the activity of other DDR components.

Though PPM1D-mutant tumors appear to be relatively susceptible to irradiation already, Akamandisa et al. demonstrated that the PPM1D inhibitor GSK2830371 could increase radiosensitivity of PPM1D-mutant tumors even further *in vitro* and *in vivo*, supposedly by restoring the activation of p53 (23). Corroborating these findings, inhibition of PPM1D has been reported to increase radiosensitivity of PPM1D-mutant cells by impairing the HDR DNA-repair pathway through reactivation of p53 (24).

The studies discussed above imply that loss of p53 activity, either directly through somatic mutations or indirectly through enhanced activity of the negative regulator PPM1D, confers radioresistance by relieving the p53-mediated brake on homology-directed repair (HDR) activity. In contrast, it has been hypothesized that radioresistance in TP53-mutant cells could be caused by RT-activated G1/S checkpoint escape rather than increased DNA-repair efficiency, an effect that may sensitize these tumors to cell cycle checkpoint inhibitors and is addressed in the following section (25). Alternatively, Deland et al. argued that the inherent radioresistance of TP53-mutant pHGG is mediated by hyperactivation of the nuclear factor erythroid 2–related factor 2 (NRF2) pathway, a key regulator of the cellular response to oxidative stress (28). Given that p53 has been reported to repress transcription of NRF2 targets, loss of p53 and subsequent activation of antioxidant pathways is likely to alter to the response to RT by reducing the level of intracellular ROS (28). Apart from the loss of function of p53, mutations in the p53 protein have been hypothesized to contribute directly to radioresistance by gain-of-function variants. For instance, knockdown or inhibition of mutant p53 has been reported to increase radiosensitivity of TP53-mutant cells (25, 43). Of note, any putative correlation in these studies between radiosensitivity and TP53 mutations may be confounded by co-occurring mutations. For instance, H3-K27M pHGG cells that, besides a TP53 mutation, harbor a mutation in the SWI/SNF chromatin-remodeling protein ATRX were shown to have an intermediate radiosensitivity with respect to other TP53-mutant and -wildtype cells (25). Indeed, loss of function ATRX mutations impair the NHEJ DNA-repair pathway, which likely reduces the repair efficiency of RT-induced DSBs (74). As research on this topic is conflicting, the exact interplay between TP53- and PPM1D mutations and radiosensitivity remains to be elucidated. Nonetheless, TP53 and PPM1D alterations appear to affect radiosensitivity differently, thereby emphasizing the necessity to distinguish between TP53- and PPM1D-mutant tumors in preclinical and clinical studies that use RT.

## Cell Cycle and GSCs

Cell cycle checkpoints play a critical role in sensing DNA damage and consequently mobilizing DNA-repair proteins, as well as halting cell cycle progression to allow time for DNA-repair (75). In general, the absence of these checkpoint kinases or downstream components causes continued cell division in the presence of DNA damage, typically leading to mitotic catastrophe and cell death (75). To improve radiosensitivity in tumor cells, researchers have tried to mimic such events by abrogating cell cycle checkpoint activity as a possible therapeutic strategy in various cancer types, including pHGG (25–32). Of note, this strategy may be of particular interest in pHGG that already possess aberrations in cell cycle checkpoints, such as TP53 mutations, as these tumor cells heavily rely on the remaining checkpoints to repair RT-induced DNA damage (19, 75). Furthermore, various studies indicate that pHGGs contain a considerable number of quiescent glioma stem cells (GSCs) intrinsically resistant to RT due to constitutive activation of cell cycle checkpoints and associated high DNA-repair efficiency (76, 77). Therefore, checkpoint inhibitors are also hypothesized to improve the efficacy of RT in pHGG by promoting re-entry of quiescent GSCs into the cell cycle (26, 78, 79). Importantly, this would not only improve the response to RT but also prevent repopulation of the tumor volume after cessation of treatment. Using patient-derived H3-K27M GSCs, one study revealed that this radiosensitization strategy is indeed specifically effective in a TP53-mutant background by demonstrating that shRNA-mediated inhibition of the checkpoint kinases ATM and CHK1 is synthetic lethal with RT in TP53-mutant but not TP53-wildtype cells (25). This synergistic anti-tumor effect with RT could also be achieved with the CHK1 inhibitor prexasertib. Inhibition of CHK1 in the absence of p53 simultaneously abrogated RT-activated G1/S and G2/M checkpoints, thereby enforcing replication in the presence of DNA damage. In contrast, TP53-wildtype cells could not be sensitized to RT by CHK1 inhibition as they remained blocked in G1. Corroborating these findings, deletion of the ATM locus has been reported to increase survival of genetically engineered mice with TP53-deficient but not TP53-wildtype brainstem gliomas following RT (28). Thus, although TP53 alterations appear to be correlated with radioresistance, they seem to evoke a specific vulnerability to the combination of RT and ATM/CHK1 inhibitors, which increase radiosensitivity by abrogating RT-induced cell cycle arrest (**Figure 2**).

In addition to the vulnerability of TP53-mutant glioma cells to ATM/CHK1 inhibitors, Werbrouck *et al.* identified a synthetic lethal interaction between RT and knockdown of the checkpoint kinases WEE1 and polo-like kinase 1 (PLK1) in TP53-mutant H3-K27M cells (25). In pHGG, both WEE1 and PLK1 are attractive therapeutic targets that are specifically overexpressed in these tumors (77). WEE1 is a checkpoint kinase that is activated by CHK1/2 and executes the cell cycle arrest at G2/M following DNA damage (75). Corresponding to this function, various studies reported that inhibition of WEE1 by the small molecule inhibitor adavosertib (MK1775/AZD1775) attenuates RT-induced cell cycle arrest and impairs repair of RT-induced DNA damage prior to entering mitosis, resulting in increased cell death *in vitro* and *in vivo* (29, 30). Of note, these effects were observed in H3-wildtype, H3-K27M, and H3-G34R/V tumors. In contrast to the preferential sensitivity of TP53-mutant cells mentioned above, Mueller et al. noticed no difference in the degree of radiosensitization based on TP53 mutational status (30). To explain this discrepancy, the authors argued that inhibition of WEE1 may directly increase DNA damage irrespective of its effect on the cell cycle, although the mechanism behind this is yet unclear (30).

Corroborating the synthetic lethality of PLK1 inhibition, Amani et al. demonstrated the radio-enhancing effect of inhibiting PLK1 in H3-K27M pHGG cells with the small molecule inhibitor volasertib (31). PLK1 is a checkpoint kinase that is inactivated by CHK1/2 following DNA damage and so inhibition of PLK1 typically leads to cell cycle arrest (80). These observations suggest that inhibition of PLK1 is associated with a different radiosensitizing mechanism than described for CHK1 and WEE1. Since PLK1 also regulates the separation of chromosomes during mitosis, it may be hypothesized that inhibition of PLK1 increases radiosensitivity evoking mitotic catastrophe. Other checkpoint kinases that regulate chromosome segregation and have also been discovered as potential radio-enhancing targets are BUB1 and BUBR1, which are part of the Budding Uninhibited by Benzimidazole (BUB) and the Mitotic Arrest Deficient (MAD) gene families of mitotic spindle checkpoints (32). In this study, inhibition of BUB1 and BUBR1 was associated with an increased formation of micronuclei, which reflects the presence of chromosomal damage, suggesting that the absence of mitotic spindle checkpoints may indeed evoke catastrophic mitotic events following RT.

The radiosensitizing abilities of volasertib may also be explained by the difference in radiosensitivity between cell cycle phases, with cells being most sensitive in G2 and M, less sensitive in G1, and least sensitive in S-phase (20). Radioresistance in the S-phase is associated with an elevated level of DNA synthesis, repair enzymes and antioxidants (20). Cells in the G2/M phase are known to be more sensitive to irradiation because there is less time for repair before chromosome segregation takes place (20). Therefore, agents that can maintain cells in radiosensitive phases (i.e., PLK1 inhibitors) or eliminate those cells in radioresistant phases are likely to cooperate with RT for enhanced efficacy (21). Surprisingly, Barton et al. demonstrated that radiosensitivity could be increased by arresting pHGG cells in G1 phase with the cyclin-dependent kinases 4 and 6 (CDK4/6) inhibitor palbociclib (**Figure 2**) (33). Notably, CDK4/6 inhibitors may be particularly effective in pHGG, which frequently harbor amplified CDK4/6 loci. Also, the expression of the cyclin-dependent kinase inhibitor p16 is typically repressed in H3-K27M tumors, which has been shown to confer susceptibility to CDK4/6 inhibition (77, 81). However, the mouse DMG models used by Barton et al. contain a genomic deletion of the Ink4-ARF locus, which are not found in DMG patients and may cause a specific susceptibility to CDK4/6 inhibitors (33).

Finally, in addition to the indirect targeting of GSCs through cell cycle checkpoints, others suggest that radiosensitivity can also be increased by directly inhibiting the stem cell-like phenotype of pHGG. For instance, inhibition of the NOTCH pathway, which is essential for maintaining stem cell-ness, with the γ-secretase inhibitor MRK003 has been shown to enhance RT-induced apoptotic cell death of H3-K27M pHGG cells (34). This study also demonstrated increased NOTCH pathway activity in primary pHGG samples and *in vitro* models, signifying NOTCH as a potential therapeutic target and suggesting that inhibiting this pathway may selectively radiosensitize the GSCs without impacting the radiosensitivity of adjacent normal tissue. Taken together, interfering with the cell cycle has yielded promising results on a preclinical level. However, it remains unclear to what extent either stimulation or abrogation of cell cycle progression is needed to maximize radiosensitivity.

## DNA Damage Repair and ROS

**Figure 3:**
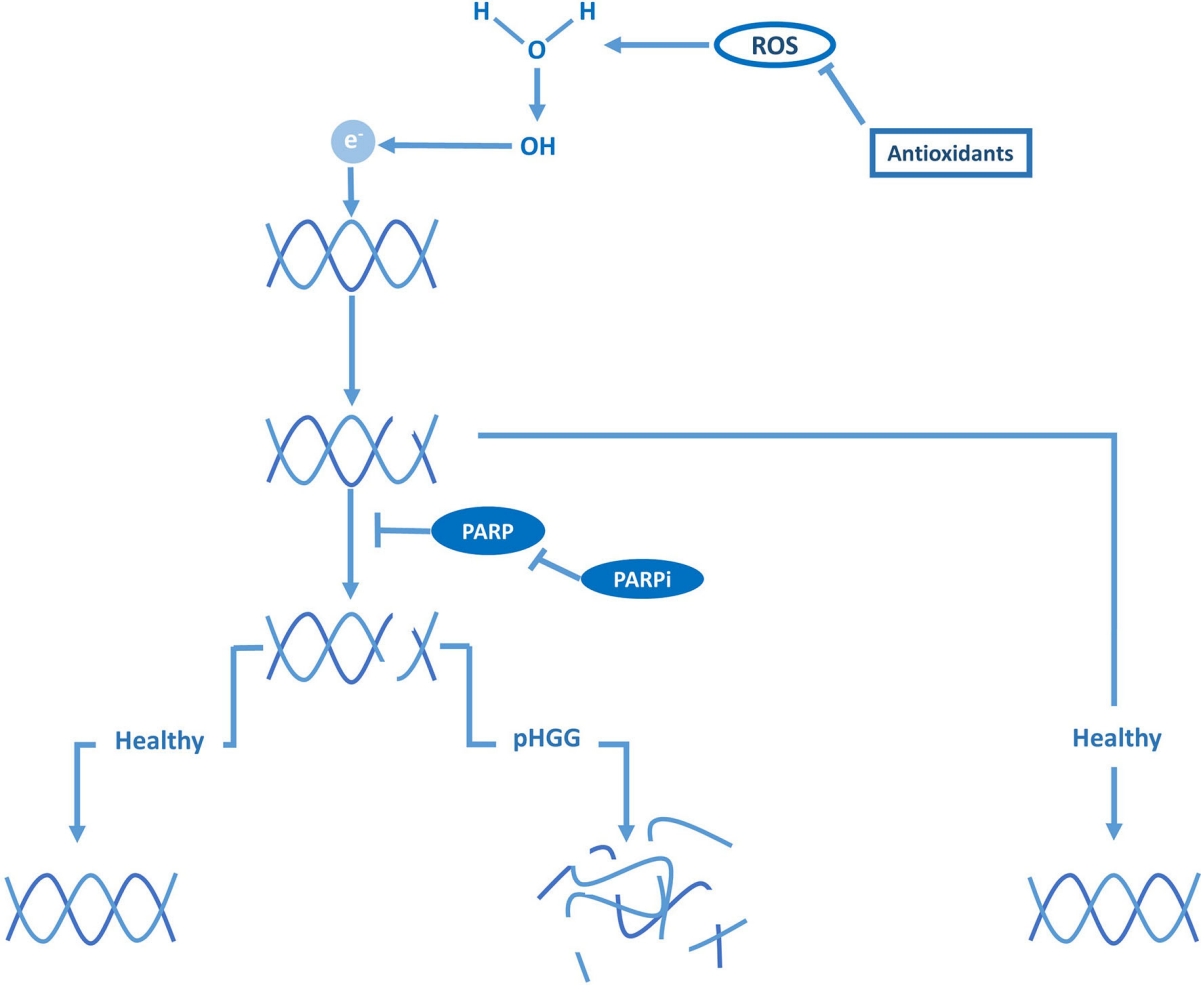
DNA damage repair and PARP are vital against ROS-induced DNA breaks in pHGG.

While the previous sections argue for the indirect targeting of DNA damage repair activity through cell cycle checkpoints, others indicate that radiosensitivity can be increased by directly blocking DNA damage repair (35, 36, 82). Tumors characterized by a high prevalence of defects in DNA-repair pathways, like pHGG, are thought to be particularly sensitive to DNA-repair inhibitors following RT, since they have become highly dependent on a few remaining DNA-repair systems (19, 75, 77). The poly ADP-ribose polymerase (PARP) enzymes, which are essential for recruiting the DNA-repair machinery to RT-induced DNA strand breaks (**Figure 3**), are especially interesting therapeutic targets as they are often overexpressed in pHGG and are thought to be predictive for prognosis (77). Several preclinical studies reported that radiosensitivity of pHGG could be increased *in vitro* and *in vivo* by inhibiting PARP activity (35, 36). These studies also demonstrated that inhibition of PARP enhances radiosensitivity by causing persistence of RT-induced DNA damage. Again, these effects were observed in H3-wildtype, H3-K27M, and H3-G34R/V tumors. The combined treatment of RT and the PARP inhibitor veliparib has also been tested in a phase I/II clinical trial; however, in contrast to the preclinical success, this study did not demonstrate a clinical benefit compared to RT alone (48). Of note, Chornenkyy et al. compared the PARP inhibitors olaparib, niraparib, and veliparib *in vitro* and demonstrated that only olaparib, niraparib, but not veliparib, were able to reduce tumor cell growth, while all inhibitors effectively inhibited PARP activity (36). Niraparib and olaparib, but not veliparib, have a dual mechanism of action by both inhibiting PARP activity and inducing the formation of cytotoxic PARP1–DNA damage complexes, suggesting a possible explanation for the low efficacy of veliparib in the clinic (36). However, limited BBB penetration of these compounds might be the main limiting factor toward clinical efficacy, which is often overlooked.

As an alternative explanation for the poor efficacy of veliparib, Versano et al. demonstrated that veliparib increases Mut-T homolog 1 (MTH1) expression, an antioxidant that protects against oxidative stress and DNA damage by hydrolyzing oxidized nucleotides (37). Consistent with this protective effect, inhibition of MTH1 by siRNAs or the small molecule inhibitor TH588 was shown to increase the anti-tumor effect of veliparib in both H3-wildtype and H3-G34R/V pHGG cells. These results imply that the potency of PARP inhibitors can be enhanced by neutralizing antioxidants and through sufficient oxidative stress. In agreement with these observations, Versano et al. further demonstrated that histone deacetylase (HDAC) inhibitors, which are known to promote oxidative stress, increase the anti-tumor effect of veliparib. Moreover, they demonstrated that MTH1 inhibition enhances radiosensitivity by exacerbating DNA damage, suggesting that neutralizing antioxidants may not only improve the efficacy of veliparib as monotherapy but also as a radiosensitizer. Others suggest that the efficacy of veliparib as a radiosensitizer may also be enhanced by blocking additional DNA-repair pathways. For example, Wang et al. demonstrated that inhibition of PPM1D sensitizes PPM1D-mutant (H3-K27M) pHGG cells to PARP inhibitors by synergistically impairing DSB repair, which also enhanced sensitivity to RT (24). Taken together, these observations imply that PARP inhibitors should not be disregarded despite the initial discouraging results of veliparib in clinical trials and that re-evaluation may be warranted. Moreover, the latter study would advocate for using a particular combination of radiosensitizers in a specific subgroup (i.e., PPM1D-mutant pHGG) rather than using a single radiosensitizer in an unstratified group of patients, as has been the case in clinical trials at large.

The studies described above suggest that other antioxidant inhibitors may also function as radiosensitizers (**Figure 3**). In pHGG, this proposition has been studied in phase I and II clinical trials with motexafin gadolinium, an inhibitor of thioredoxin and ribonucleotide reductases, and etanidazole, an inhibitor of glutathione S-transferase (49–51). Although these compounds could be safely administered in combination with RT, these trials did not advance further than phase II due to a lack of superior efficacy over RT. Nonetheless, as indicated above, these inhibitors may still boost the radiosensitizing effect of other strategies, suggesting that the full potential of exploiting oxidative stress as a radiosensitizing strategy is yet to be uncovered. Taken together, although compounds targeting DNA-repair and ROS pathways have not yet proven to be successful as radiosensitizers in pediatric glioma patients thus far, using these compounds in the right combination may be a promising radiosensitizing strategy against certain pHGG subgroups.

## MAPK/PI3K

**Figure 4:**
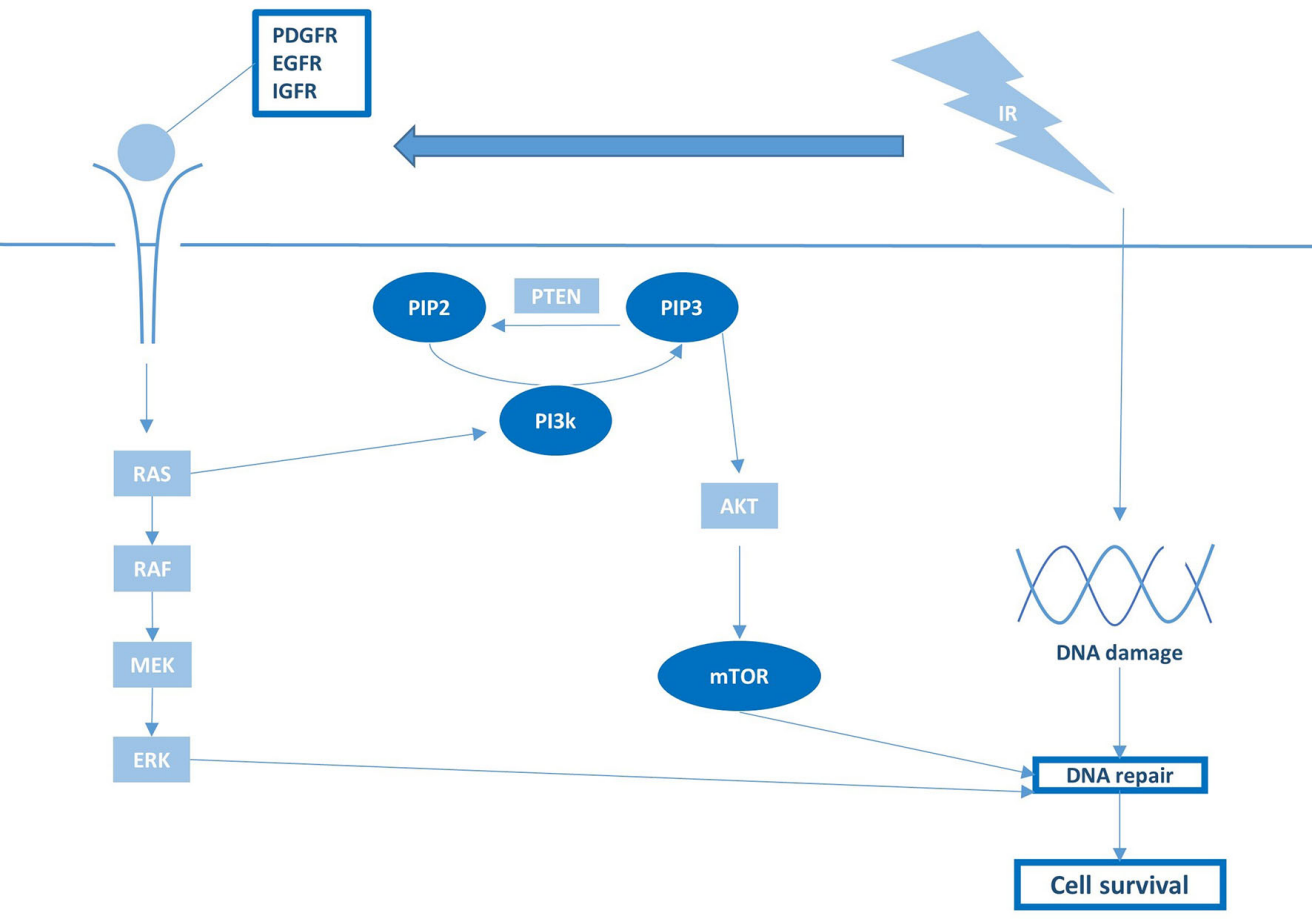
Growth factor receptor activation and downstream PI3K/mTOR signaling are pivotal regulators of RT sensitivity and pHGG survival.

As for most cancers, mitogenic MAPK and PI3K signaling pathways are often constitutively active in pHGG due to mutations or gene amplification in core components or upstream proteins, such as receptor tyrosine kinases (RTKs) (77). When tissue injury and cell loss occurs following radiation, these mitogenic signaling pathways are usually further activated, which leads to an enhanced proliferation rate and repopulation of the tumor volume after treatment (19). Moreover, these pathways stimulate the repair of RT-induced DNA damage by regulating the expression of DDR components (19). As such, hyperactive MAPK and PI3K pathways typically elevate the baseline DNA damage repair capacity of pHGG tumors and thereby contribute to their radioresistant phenotype (**Figure 4**). To reduce both DNA-repair efficacy and repopulation following radiotherapy, various studies have investigated inhibiting upstream or downstream MAPK and PI3K components in combination with irradiation (38–41). One study demonstrated that radioresistance in pHGG correlates to overexpression of the RTK insulin-like growth factor receptor (IGF-1R), which, in turn, correlates with a worse prognosis in pHGG patients (38). Furthermore, they demonstrated that inhibition of IGF-1R with the small molecule inhibitor BMS-754807 enhances radiosensitivity of H3-wildtype and H3-G34R/V pHGG cells by impairing the repair of RT-induced DNA damage. Likewise, several studies demonstrated that inhibition of the mammalian target of rapamycin (mTOR) complex, a downstream effector of IGF-1R, increases radiosensitivity of H3-wildtype and H3-K27M pHGG cells (39–41). However, although mTOR acts downstream of IGF-1R, these mTOR inhibitors did not appear to recapitulate the increase in DNA damage that was observed for IGF-1R inhibition. Miyahara et al. demonstrated that the radiosensitizing effects of mTOR kinase inhibitor TAK228 was instead due to a downregulation of anti-apoptotic proteins (39). In contrast, Agliano et al. reported that the PI3K/mTOR inhibitor NVP-BEZ235 increases radiosensitivity by abrogating RT-induced G2/M arrest rather than affecting apoptosis or DNA-repair efficiency (41). Therefore, further studies are required to elucidate whether shared or unique radiosensitizing mechanisms underlie these observations and which target or mechanism in these pathways is critical for improving radiosensitivity.

Although targeting MAPK and PI3K through inhibition of RTKs appears a promising radiosensitization strategy on a preclinical level (**Figure 4**), the RTK inhibitors tested to date in pHGG clinical trials uniformly failed to improve prognosis over RT alone (52–64). As many small molecules developed as anti-cancer drugs have historically been selected for their inability to pass the BBB to minimalize neurological side effects, the failure of these inhibitors in pHGG patients may be attributed to inadequate drug delivery. In this regard, it is worth noting that clinical trials nowadays increasingly incorporate compounds or vehicles with good brain penetration and distribution. As one example, a phase I trial has recently been initiated with a novel brain-penetrant PI3K/mTOR inhibitor GDC-0084 in newly diagnosed DMG (NCT03696355). While awaiting the results of these studies, the true clinical feasibility and efficacy of this radiosensitization strategy remain elusive.

## Epigenome

**Figure 5:**
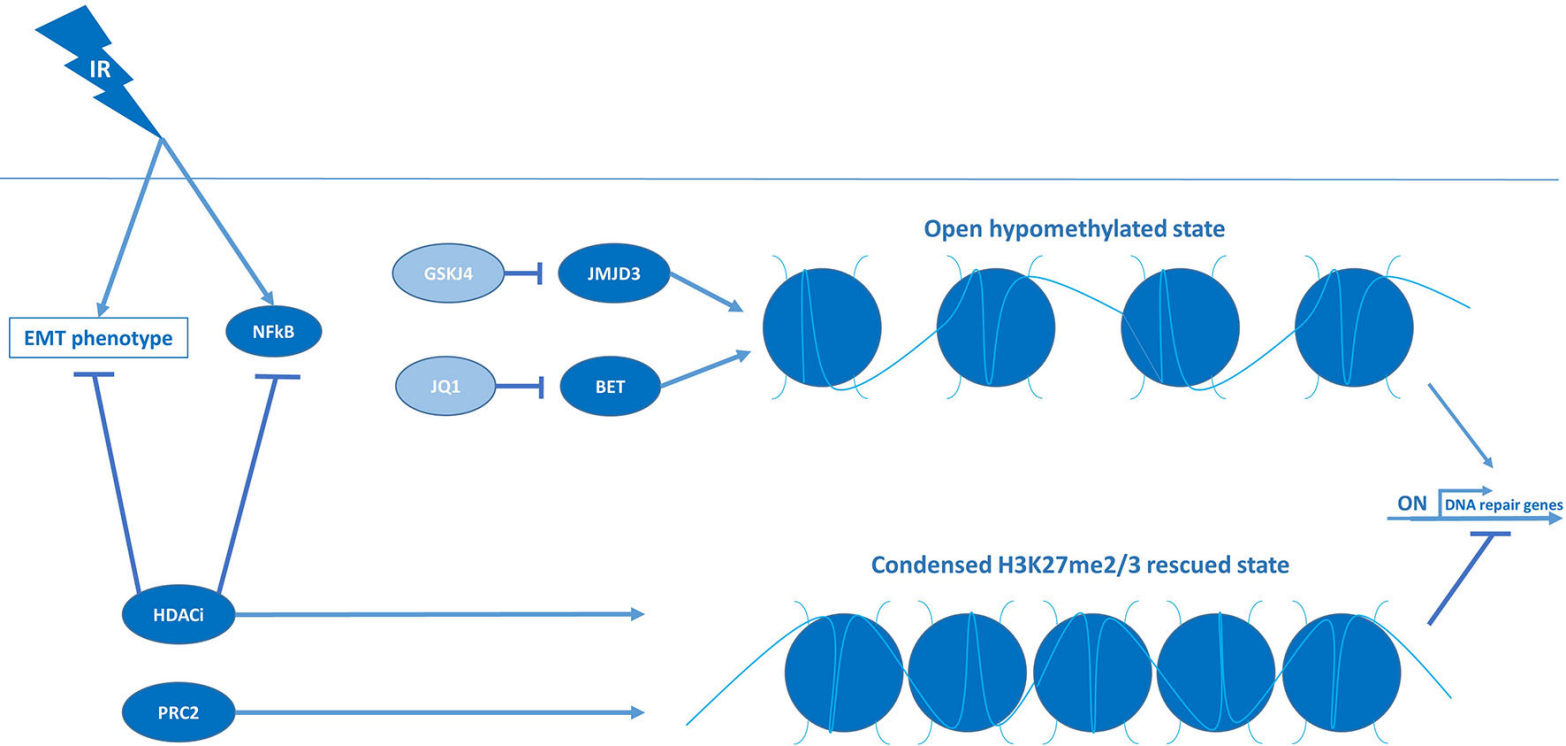
Condensed chromatin structures suppress DNA-repair machineries and induce RT sensitivity in pHGG.

Based on the high prevalence of histone H3 mutations in pHGG, and their consequences for chromatin remodeling and gene transcription, reversing the aberrant methylation/acetylation balance in these tumors using epigenetic modifiers has been extensively investigated over the last decade as a possible therapeutic strategy (1). One strategy has been directed at restoring di- and trimethylation of H3-K27 (H3-K27me2/3) in H3-K27M tumors by inhibiting the lysine 27-specific histone demethylase jumonji domain containing-3 (JMJD3). While JMJD3 inhibitors show promising anti-tumor effects as monotherapy, the JMJD3 inhibitor GSK-J4 has also been reported to increase radiosensitivity *in vitro* and *in vivo*, specifically in H3-K27M tumors (42). In those tumors, GSK-J4 treatment impaired the repair of RT-induced DNA damage by reducing the expression of DNA-repair genes (**Figure 5**). Furthermore, GSK-J4 was shown to block DNA-repair by arresting the cell cycle in early S phase and, consequently, excluding the HDR pathway that is only active in late S/G2. In contrast, GSK-J4 did not affect the expression of repair genes and did not improve radiosensitivity in H3-wildtype tumors (42). Nikolaev et al. corroborated these findings in H3-K27M pHGG and demonstrated that the radiosensitizing effect of GSK-J4 could be enhanced in TP53-mutant cells by adding APR-246, an agent that forms covalent bonds with mutant p53 and neutralizes the protein (43). Since GSK-J4 treatment restores H3-K27me2/3, these findings indicate that reversing the hypomethylation phenotype of H3-K27M tumors is not only cytotoxic but may also improve the response to RT.

Another consequence of H3-K27M mutations is an increase in H3-K27 acetylation (H3-K27ac), resulting in an open chromatin structure and subsequent transcriptional activation at these genomic loci (77). Although it seems counterintuitive, a therapeutic strategy that has been investigated is to aggravate this hyperacetylation state using HDAC inhibitors (77). By increasing histone acetylation, these inhibitors appear to rescue the hypomethylation phenotype indirectly and, as a result, reduce tumor growth (**Figure 5**) (77). HDAC inhibitors have even been proposed to “detoxify” H3-K27M-induced inhibition of PRC2, but whether this is clinically relevant remains to be determined as various studies indicate that the response to HDAC inhibition is unrelated to histone mutational status (1, 83). Concerning radiosensitivity, HDAC inhibitors have been reported to reduce the expression of checkpoint kinases and DNA-repair genes and, as such, have been proposed to also function as radiosensitizers. For instance, Pal et al. showed that a dual inhibitor of HDAC and PI3K, fimepinostat, sensitized a large panel of *in vitro* and *in vivo* pHGG models to irradiation by downregulating the checkpoint kinases WEE1 and CHK1 (46). Like checkpoint inhibition, the reduced expression of WEE1 and CHK1 resulted in abrogation of RT-induced G2/M arrest and enforced replication in the presence of DNA damage. Additionally, they demonstrated that fimepinostat induces G1 arrest, which, similar to GSK-J4, is known to inhibit the repair of DNA damage by excluding the HDR pathway. Fimepinostat was also shown to block the repair of RT-induced DNA damage by downregulating genes essential for HDR and NHEJ. To explain these observations on a mechanistic level, Pal et al. further demonstrated that fimepinostat blocks RT-induced expression and nuclear localization of the nuclear factor kappa-B (NFkB) transcription factors, thereby impairing gene expression of this vital NFkB survival mechanism (**Figure 5**). In line with this observation, knockdown of NFkB recapitulated the radiosensitizing effect of fimepinostat, suggesting that NFkB inhibitors may also be of value in combination with RT in pHGG. Furthermore, it is worth noting that these effects were observed in both H3-wildtype, H3-K27M, and H3-G34R/V tumors, suggesting that at least some of the radiosensitizing effect of HDAC inhibition is unrelated to histone mutational status. In line with these findings, the HDAC inhibitor abexinostat has been shown to increase radiosensitivity of H3-wildtype and H3-G34R/V cells by reducing the expression of genes essential for HDR and NHEJ (44). Taken together, these findings advocate for the use of HDAC inhibitors as radiosensitizers and the broad applicability of these compounds.

In addition to their effects on DNA-repair, HDAC inhibitors are of interest in pHGG due to their ability to reverse epithelial-to-mesenchymal transition (EMT), a process in which epithelial cells adopt a mesenchymal phenotype by loss of cell-cell adhesion and acquisition of migratory properties (16). This migratory phenotype, which is stimulated by RT, is hypothesized to allow the tumor cells to escape from the irradiated area, thereby evading the treatment (9, 16). This transition is also believed to be responsible for the induction and maintenance of stem cell characteristics and, consequently, a higher radioresistant phenotype (16). Recently, we demonstrated that the HDAC inhibitor panobinostat can reverse the EMT phenotype and that this effect can be enhanced by simultaneously inhibiting the growth factor receptor AXL, a putative driver of EMT (45). They further demonstrated that combined treatment with the AXL inhibitor BGB324 and panobinostat downregulates the expression of genes associated with stem cell maintenance and DNA-repair. This reversal of the mesenchymal, stem cell-like, therapy-resistant phenotype of H3-K27M pHGG cells resulted in a synergistic anti-tumor effect and a robust sensitization to RT *in vitro*. Notably, while panobinostat was observed to function as a radiosensitizer alone, it could not prevent tumor regrowth. However, the addition of BGB324, having no significant radiosensitizing effect on its own, produced robust triple synergy in combination with panobinostat and RT and completely abolished tumor growth. These findings suggest that a combinatory approach may be necessary to improve radiosensitivity sufficiently. In line with this hypothesis, the HDAC inhibitors tested in combination with RT in clinical studies, demonstrated encouraging response rates but have not been able to significantly improve survival compared to conventional treatment (65–69). Taken together, HDAC inhibitors may considerably enhance the response to RT by reversing EMT, although a combinatory approach may be necessary to achieve a significant effect.

Another therapeutic approach related to the H3-K27-dependent increase in histone acetylation is directed at the occupancy of the H3-K27ac sites by bromodomain and extra-terminal (BET) proteins, reader proteins that associate with acetylated histones and recruit the transcriptional machinery to initiate expression (11, 84). Displacement of BET proteins from acetylated histones is known to disrupt RNA polymerase II-mediated transcription, thereby reducing the high-level expression of oncogenes associated with H3-K27M mutations (1). Regarding radiosensitivity, inhibition of the BET protein family member bromodomain-containing protein 4 (BRD4) with the small molecule inhibitor JQ1 has been shown to markedly reduce the expression of DNA-repair genes and sustain high levels of RT-induced DNA damage in H3-K27M cells, leading to an enhanced RT effect *in vitro* and *in vivo* (47). Altogether, by affecting cell cycle checkpoints, DNA-repair, and EMT, targeting the epigenome combined with RT holds great potential for improving radiosensitivity of pHGG tumors.

## Discussion

Until a decade ago, pre-clinical research to understand the molecular characteristics of pHGG was virtually absent due to a lack of representative culture and xenograft models. Furthermore, to this day clinical treatment protocols in pHGG are often derived from trials in adult patients, and effective therapeutic options remain scarce. With the implementation of biopsy and autopsy protocols for collecting biological pHGG material, preclinical research is expanding rapidly, and our understanding of the pathobiology of these malignancies has improved tremendously. One of the most vital discoveries from these recent preclinical studies encompasses the identification of H3 mutations in about 50% of all pHGG, in major contrast to aHGG, including its correlation with age of onset, aggressiveness, and location of the tumor (15). The epigenetic deregulated nature of these tumors has been discovered to contribute to a stem cell-like, therapy-resistant phenotype, which further sets these tumors apart from their adult counterparts (9). These differences also result in a strong differential RT response between pediatric and adult glioma (85). Now that adequate pHGG *in vitro* and *in vivo* models are available and increasingly used in research, re-evaluation of radiosensitization may prove valuable for improving the standard of care for these fatal childhood brain tumors, for which this review serves as a guide.

**Figure 6:**
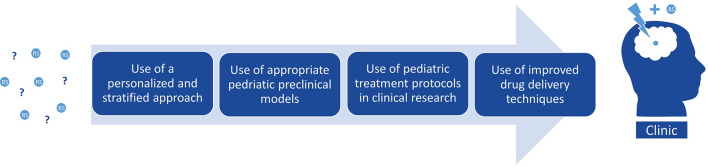
Considerations for improved clinical translation of pre-clinical radiosensitizers in pHGG.

The studies discussed in this review draw the image of an intricate balance between radiosensitivity and radioresistance in pHGG based on the mutational status of each tumor. For instance, mutations in upstream or downstream regulators within the same pathway do not necessarily phenocopy the level of radiosensitivity, as illustrated by p53 and PPM1D mutations. Furthermore, some combinations of co-occurring mutations alter the response to RT, as shown by the intermediate radiosensitivity of TP53- and ATRX-mutant cells with respect to other TP53-mutant and TP53-wildtype cells. As discussed in this review, several studies also show that the mutational status of pHGG evokes vulnerabilities to specific radiosensitizing agents. For example, checkpoint inhibitors are overall better radiosensitizers in TP53-mutant than TP53-wildtype tumors, and PPM1D inhibitors specifically radiosensitize PPM1D-mutant tumors. However, radiosensitivity is often a more complicated matter, as sufficient oxidative stress or DNA damage are often required to induce the desired RT-enhancing effect of radiosensitizers, as shown with combined antioxidant and PARP inhibitor treatment. Furthermore, sometimes drug-synergy is only effective in a specific mutational background, as with combined PPM1D and PARP inhibition in PPM1D-mutant tumors. These observations emphasize the necessity for a personalized and stratified approach (**Figure 6**) rather than applying a single radiosensitizer to an unstratified group of patients, as has been the case in the majority of clinical trials to date.

Pediatric gliomas are hallmarked by epigenetic dysregulation, often caused by H3 mutations, which widely impact tumor-behavior and emphasizes the necessity of a treatment strategy tailored to a histone-mutant or -wildtype background. However, this concept has thus far only been demonstrated with JMJD3 inhibitors in H3-K27M pHGG, possibly because epigenetic dysregulation in cancer is a relatively new field of research and still poorly understood. Since abnormal histone functioning is associated with genomic instability (especially with G34R/V mutations), compounds that impair DNA-repair or checkpoint kinases may specifically improve radiosensitivity in these histone-mutant tumors by causing an overload of DNA damage. Although most studies discussed in this review did include different histone H3-mutant and -wildtype models, differential sensitivity was not assessed in the majority of these studies, warranting further investigation.

Although representative pediatric models are pivotal in the search for effective therapy against pHGG, results from studies in adult patients or models may still be relevant. An important query that has been studied in aHGG is the relation between radiobiology and the immune system. Here it was shown that irradiation activates the immunosuppressive “cold” environment in brain tumors and triggers an abscopal effect: a phenomenon in which RT initiates an immune response that eliminates cancer cells distant from the irradiated volume (86). Given the diffuse growth characteristics of pHGG, this may represent a promising radiosensitization strategy. Immunotherapy for pediatric brain tumors is an area that is relatively unexplored in preclinical research, mainly due to the lack of immunocompetent *in vivo* models. The development of these models would allow us to study the interactions between immune cells and radiotherapy in patient-derived pHGG models *in vivo*, which may revolutionize the field of radiosensitization in pediatric brain tumors. Fortunately, novel methods have recently been published that describe the generation of spontaneous murine HGG models with or without histone 3 mutations, accompanied by somatic mutations of choice (87–89). When used within a similar mouse strain, cells from these spontaneous models can easily be used to generate xenografts in immunocompetent mice, allowing us to study the interaction between tumor-microenvironment, immune system and treatment. Furthermore, these models resemble a more realistic pathophysiology compared to many of the older inducible cancer models, which are often generated in the presence of mutations that are rarely found in those tumors.

Despite the promising radio-enhancing effects of the agents addressed in this review, a recurrent problem with small molecule inhibitors remains the limited distribution through the brain due to their inability to cross the BBB. Although the BBB is often disrupted in aHGG, the integrity of this barrier in pHGG is often more intact, especially in DMG, but also appears to have a heterogeneous representation (90). Regardless of BBB integrity within the tumor, the ability to penetrate the brain remains a prerequisite for any compound used in these diffusely growing brain tumors as tumor cells can migrate into regions of the brain with an intact BBB. In this regard, promising innovations emerged in recent years to disrupt or circumvent the BBB, like convection-enhanced delivery (CED) and sonoporation using high-intensity focused ultrasound (HIFU) combined with microbubbles (90–92). These novel strategies have only recently been developed for clinical use and will be of vital importance for the efficacy of radiosensitizers in patients (**Figure 6**). Furthermore, with the help of these brain penetrating strategies, we might want to reconsider some of the many potential RT-enhancing agents that have failed translation to the clinic since the mid-1980s.

## Conclusions

pHGGs are highly malignant brain tumors with a devastating prognosis, causing the most cancer-related deaths in children. Radiotherapy is part of the standard therapy against pHGG and often the only option to provide temporary symptom relief and a delay in tumor progression. Various preclinical and clinical studies have evaluated the potential of improving sensitivity to radiotherapy by targeting key survival or radioresistance mechanisms combined with irradiation. Although these strategies appear promising at a preclinical level, the radiosensitizers tested to date in clinical trials have not yet significantly improved survival. Nonetheless, the last decade has taught us much about the behavior, vulnerabilities, molecular characteristics, and modeling methods of pHGG. With this knowledge and access to a plethora of target-specific small molecule inhibitors, a variety of clinically relevant possibilities towards pHGG-specific radiosensitization can now be explored. Especially with the increasing availability of biological material and adequate *in vitro* and *in vivo* models, as well as the development of novel brain-penetrant agents, designing an effective radiosensitizing strategy for these fatal childhood brain tumors is at an apparent reach.

## Author Contributions 

DM and AC conceived and designed this review. DM and AC have written the manuscript. DM and IS designed and created illustrations. GK and EH acquired funding and provided supervision.

All authors have reviewed the manuscript. All authors contributed to the article and approved the submitted version.

## Conflict of Interest

The authors declare that the research was conducted in the absence of any commercial or financial relationships that could be construed as a potential conflict of interest.
